# The power of interspecific sociality: how humans provide social buffering for horses

**DOI:** 10.1007/s10071-025-01942-5

**Published:** 2025-03-12

**Authors:** Alfredo Di Lucrezia, Anna Scandurra, Daria Lotito, Valeria Iervolino, Biagio D’Aniello, Vincenzo Mastellone, Pietro Lombardi, Claudia Pinelli

**Affiliations:** 1 Department of Environmental, Biological and Pharmaceutical Sciences & Technologies, University of Campania Luigi Vanvitelli, 81100 Caserta, Italy; 2 Department of Biology, University of Naples Federico II, 80126 Naples, Italy; 3 Department of Veterinary Medicine and Animal Production, University of Naples Federico II, 80137 Naples, Italy

**Keywords:** Horses, Social buffer, Sociality, Heart rate, Isolation paradigm, Cortisol, Human-animal interaction

## Abstract

In this study, we assessed the interspecific “social buffering effect” of humans on horses, exploring how human presence influences stress responses in horses in an unfamiliar environment using the “isolation paradigm.” We examined nine Haflinger horses under two counterbalanced conditions: with a passive human stranger (*social* condition) or alone (*isolation* condition). Stress responses were assessed through cortisol measurements, heart rate monitoring, and behavioral observations. While cortisol levels significantly increased in both conditions, with no notable differences before and after the tests, heart rate data revealed a different pattern. Results indicated that stress generally decreased in both scenarios, impacting heart rate. Initially, during the first five minutes, heart rate was significantly higher in the social condition compared to isolation, but this trend reversed in the following intervals, with heart rate significantly decreasing as interaction with the stranger increased. Positive interaction between time and stranger-directed behaviors suggested the stranger’s influence on heart rate strengthened over time. Overall, these finding suggest that while cortisol data did not reflect a social buffering effect, other metrics indicated that human presence effectively reduced stress in horses after a brief adjustment period, supporting the hypothesis that horses can benefit from human presence during stress, after a short adaptation time. This study highlights the complex nature of stress responses in horses and the potential role of humans as social buffers in interspecific contexts.

## Introduction

Sociality can impose costs on individuals, heightening competition for resources and mates, facilitating disease transmission, and raising the group’s visibility to predators (Silk 2007). Conversely, living in groups provides social animals with several advantages such as decreased vulnerability to predators, improved efficiency in locating food (Silk 2007), and easier acquisition of knowledge through social learning (Krause et al. 2010; Ashton et al. 2019). When the benefits outweigh the costs, and sociality becomes an evolutionarily stable strategy (Krause and Ruxton 2002), separation from conspecifics can trigger stress responses, encompassing behavioral and physiological changes (Levine 1967). Alongside neural signals from the sympathetic nervous system, which directly prompt the adrenal glands to discharge adrenaline and noradrenaline, the involvement of the hypothalamic-pituitary-adrenal (HPA) axis in response to stressors has been long recognized (Selye 1936). In this context, research indicates that the mere presence of a social partner can hinder or dampen physiological and behavioral responses to stressors, thus aiding in improved facing with adverse experiences (Hennessy et al. 2009). Specifically, this effect works by modulating the activity of the HPA axis, resulting in reduced secretion of stress hormones like cortisol and the restoration of physiological balance (von Holst 1998). Known as “social buffering”, this well-documented phenomenon has been observed in humans and increasingly recognized in other animals, highlighting the importance of social partners in dealing with stress-inducing situations (Kikusui et al. 2006; Hennessy et al. 2009; Rault 2012; Wu 2021).

The efficacy of the social buffering effect on a subject can vary depending on several factors, including the social structure of the species, the individual history of the subject, the gender, the type and severity of the stressor, the environmental context in which the interaction occurs, and the familiarity with the social partner (Rault 2012). This phenomenon has been extensively studied within the framework of interactions among individuals of the same species and has been explored across numerous non-human animals (Kikusui et al. 2006; Hennessy et al. 2009; Hostinar et al. 2014; Kiyokawa and Hennessy 2018). However, the interspecific aspect of social buffering, where humans provide beneficial psychological effects on animals, has received comparatively less attention.

The bond between humans and domesticated animals, which fulfill diverse roles in human societies, is an area of keen interest. Dogs and humans share a distinctive psychoemotional connection facilitated by various sensory inputs, including visual (Müller et al. 2015; Albuquerque et al. 2016) and auditory cues (Albuquerque et al. 2016), as well as olfactory signals (D’Aniello et al. 2018, 2021, 2023). Therefore, it is not surprising that numerous studies have demonstrated that dogs experiencing stressful situations exhibit reduced cortisol levels after positive interactions with humans (Hennessy et al. 1997; Menor-Campos et al. 2011; Shiverdecker et al. 2013; Dudley et al. 2015; Sandri et al. 2015; Willen et al. 2017; Gunter et al. 2019; Buttner et al. 2023).

One of the most effective protocols for inducing stress responses in social species is the isolation paradigm, where a subject is separated from its peers and placed alone in an unfamiliar environment. In a study employing the isolation paradigm, dogs left alone in a novel indoor setting exhibited a significant increase in cortisol (and corticosterone) levels, which was notably attenuated when a familiar person was present in the same environment (Tuber et al. 1996). Subsequent research indicated that even unfamiliar humans can act as social buffers for dogs, regardless of whether the dogs were born and raised in shelters with limited social interaction with humans (Pinelli et al. 2023). A comparative study demonstrated that goats separated from their social groups and placed in a new environment, unlike dogs, did not experience stress mitigation when humans were present. Indeed, some goats choosing to interact with the experimenters during the test did experience the human social buffering effect similar to dogs (Scandurra et al. 2024). This suggests that the factors influencing the benefits of social buffering are complex and extend beyond the domestication process, highlighting the importance of individual variability and willingness to engage in social interactions with humans.

Horses are among the domesticated species inhabiting the anthropomorphic niche, sharing goals and environments with humans. They have been domesticated for approximately 6000 years (Warmuth et al. 2012) and have since been utilized by humans for various purposes, including sports, companionship, and as working animals (Merkies and Franzin 2021). This makes horses worthy subjects for studying the potential social buffering effect provided by humans. Social separation in horses is known to trigger stressful responses (Lundblad et al. 2021). In this context, interspecific studies have shown that the effectiveness of social buffering is influenced by the type of stimulus rather than the familiarity of the companion (Ricci-Bonot et al. 2021). Such studies reported that, while both familiar and unfamiliar companions reduced behavioral responses to a mildly frightening stimulus like a static ball (i.e., novel object test), they did not have the same effect during a more intense stimulus, such as an opening umbrella. In a follow-up study, Ricci-Bonot et al. (2023) investigated whether a visual substitute, a poster of a relaxed horse’s face, could act as a social buffer in stressful situations. Horses were tested with a novel object and an umbrella opening test, measuring behavioral and physiological responses. The poster significantly reduced behavioral responses in the novel object test but had no significant effect on responses during the umbrella test. This suggests that the effectiveness of social buffering may depend on the nature of the stressful event. Additionally, Mills and Riezebos (2005) noted that stereotypic behaviors in horses could be reduced when exposed to a life-size poster of a horse’s head, although it remains unclear if this effect is due to social buffering or simply visual distraction.

Like dogs (Miklósi and Topál 2013), horses are believed to share a special psychoemotional relationship with humans (Scopa et al. 2019), likely stemming from their close proximity in shared working roles. Horses can individually recognize a familiar person and appeared to be more relaxed while physically interacting with some familiar handlers compared to unfamiliar ones (Scopa et al. 2020). However, no differential benefits were observed when comparing passive unfamiliar and familiar figures (Scopa et al. 2020). Similarly, other studies have not found compelling evidence of a differential effect on stress responses in interactions with strangers versus familiar figures (Hawson et al. 2010; McLean and McGreevy 2010). Conversely, Marsbøll and Christensen (2015) reported that familiarity reduced behavioral fear responses during handling tests, though this effect did not extend to interactions with unknown handlers. Given these conflicting findings regarding the role of familiarity in horses, we opted to use a stranger as a potential social buffer to avoid introducing an uncontrolled confounding factor. Furthermore, our previous research on dogs demonstrated that even unfamiliar humans could act as effective social buffers (Pinelli et al. 2023). This choice not only helps to mitigate the potentially masking effects of familiar handlers on stress responses but also aligns better with real-life stressful scenarios that horses may encounter. Our research was carried out on a farm where horses regularly interact with unfamiliar humans, and the farmers take great care to minimize negative interactions with strangers. We investigated the effect of human presence within the context of the isolation paradigm. The experimental setup involved introducing a passive stranger into the environment. Unlike dogs, horses, as prey animals, may perceive humans as potential predators, triggering stress responses during interactions with unfamiliar individuals. However, considering that the horses selected for this study have positive experiences with unfamiliar people, along with the longstanding cooperative bond between horses and humans, we expect that the presence of an unknown person may still exert a beneficial influence on their emotional responses when they are in moderately stressful conditions, such as in the isolation paradigm.

## Materials and methods

### Animals

The research was conducted at the horse breeding “Meola Alessandro”, a farm situated in Morcone, Campania, Italy. Data collection occurred over two experimental sessions conducted in September and October 2023. Eleven Haflinger horses (*Equus ferus caballus*) were included in the experiments, comprising 5 females and 6 males, with a mean age of 4.5 ± 4.4 years (ranging from 2 to 17 years). However, due to technical issues (see below), the statistical analysis was conducted on 9 horses (4 females and 5 males; mean age of 5.0 ± 4.8 years). The horses were kept in semi-extensive breeding conditions, with ample freedom to roam during the day, and individually stabled in 4 × 4 m boxes at night. Their diet consisted of grazing supplemented with hay during dry periods and scarcity of grass. In winter, they were fed hay, crushed barley, and oats, with feeding occurring at 01:00 pm.

The horses had the opportunity to leave their boxes in the daytime forming three distinct groups: one comprising broodmares, including pregnant or lactating mares; another consisting of foals older than one year but not sexually mature; and a group of adult males (stallions and geldings). Breeders emphasized handling and husbandry practices aimed at fostering positive social interactions within and between species from an early age.

All horses interacted daily with humans during routine care and periodically for veterinary checks, excursions, and training activities. Socialization sessions with humans focused on gentle handling to enhance the horses’ adaptability and confidence with unfamiliar individuals. As a result, breeders prioritized actions aimed at developing welfare-centered human-horse interactions, leading to horses generally displaying confidence with strangers and minimal fear responses. The sessions are held on weekends, when people visit the farm to go horseback riding, except during periods of bad weather.

Horses for the study were randomly selected from the group, excluding animals under 2 years of age, as well as pregnant or lactating mares.

### Experimental room (Fig. 1)

**Fig. 1:**
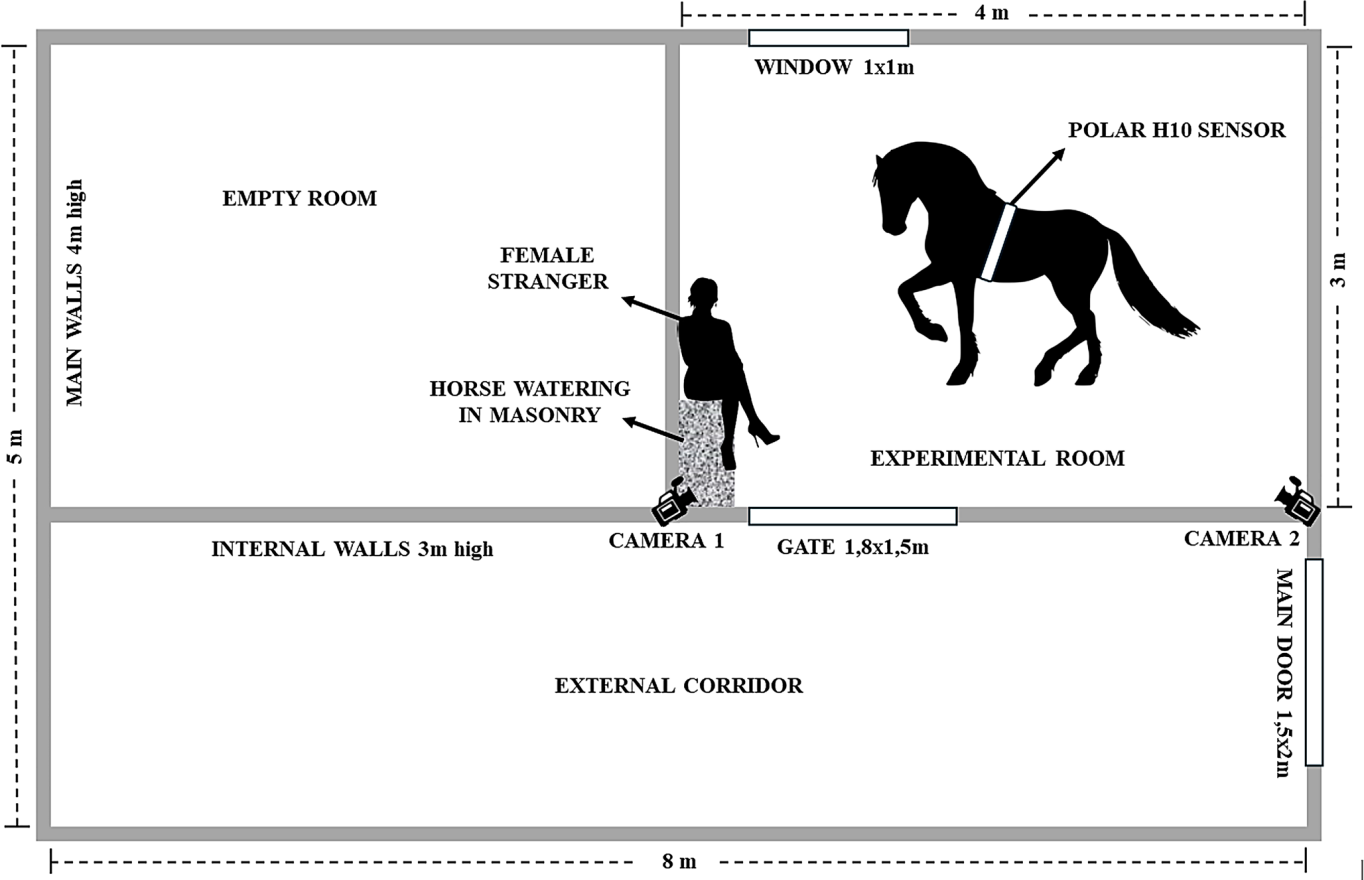
Experimental room. The figure illustrates the features of the experimental room and pertains to the “*social*” condition of the experiment, where a passive stranger was present with the animal during testing. In the “*isolation*” condition, the animal is alone in the same room

The experiments were carried out in a small stable measuring approximately 8 × 5 m in area and standing 4 m tall, about 200 m away from where horses were normally kept. Within this stable, two rooms were delineated by the primary walls on two sides and with internal walls reaching a height of 3 m. The room nearest to the entrance, spanning about 4 × 3 m, was selected for the experiments. The primary entrance to the stable was an iron door, while the entrance to the internal room was a gate approximately 1.80 m high and 1.5 m wide, which was covered with a cloth to limit the horses’ visual access to the external area. The room also featured a window, kept closed, measuring 1 × 1 m, situated at a height of 2 m from the ground inside the room and at ground level externally. A researcher was assigned to monitor and address any visual or auditory disruptions caused by the hosts of the structure. The experiments were recorded using two strategically positioned cameras (Sony^®^ HDR CX115 and Sony^®^ HDR-PJ260VE) which were placed in two adjacent corners above the walls. As a result of limited luminosity, the room had to be artificially illuminated by a light fixture affixed to the center of the ceiling. One of the cameras was equipped with a wide-angle lens. Before each test, the room underwent cleaning procedures, including the removal of feces using a shovel and the application of abundant water from a fountain utilizing a rubber hose. At the time of the experiment, the stable had been used as a storage area for many years and was cleared out and cleaned for the experiments. It was unfamiliar to the horses from the facility.

### Procedure

After selecting the horse, it was gently led in proximity of the main entrance of the stable by the horse keeper where the animal was equipped with the heart rate monitoring system. Once the heart rate system was effective a veterinarian promptly collected blood samples. Immediately afterward, the horses were led inside the stable and then into the experimental room by the keeper. Subsequently, the keeper exited the experimental room, closing the door behind them, and then left the stable, closing the main door. The test lasted for 15 min, a duration sufficient to induce significant changes in blood cortisol levels following a stressing event (Peeters et al. 2011). Upon the testing time expired, the keeper returned to retrieve the horse from indoors and led it outdoors for the second blood sampling.

Each animal underwent two conditions: “*isolation*” and “*social*” on 2 consecutive days. During the isolation condition, the horses were left alone in the room for the duration of the test. In contrast, during the social condition, a female stranger was present in the room. These two conditions were counterbalanced to minimize any order effects. The stranger settled on a stone horse watering system positioned in a corner of the room, maintaining a passive behavior even upon eventual horses’ solicitation, also avoiding making eye contact with them. Ensuring the stranger’s passivity was crucial to maintain consistency and standardization in the experiment, given the challenge of controlling human activity levels, which could unpredictably influence horse behavior and complicate the reproducibility of research findings. Moreover, it is suggested to apply the concept of social buffering when a subject benefits from the mere presence of a conspecific, without direct actions aimed at alleviating another subject’s stress (Wu 2021). The stranger was a highly experienced horse rider and trainer, confident in handling horses and capable of managing any potential threats with minimal movements. Moreover, to exclude any potential effects linked to familiarity, she had no prior contact with the horses included in the current experiments.

### Blood sampling and cortisol assay

Experiments were scheduled in the afternoon, spanning from 14:00 to 18:00. This timeframe was chosen because previous research indicates that cortisol levels in horses during this period are intermediate between the peak level observed at 10:00 and the lower levels seen at 22:00 (Bohák et al. 2013), thus allowing to mitigate potential roof and floor effects. Furthermore, cortisol levels in horses tend to exhibit relative stability in the afternoon (Irvine and Alexander 1994). Additionally, to further minimize any bias related to the cortisol circadian cycle, each horse underwent testing at the same time on both days.

Blood samples were collected by jugular venipuncture. This procedure was closely monitored in real-time through the heart rate monitoring system. Remarkably, the horses’ heart rate remained practically unchanged during the blood sampling, with observed changes ranging between 0 and ± 2 units. This minimal (or absent) fluctuation suggests that the blood sampling procedure was not stressful for these horses. Additionally, it was proved that blood sampling (by catheterization) in horses did not show significant variation in blood cortisol after one hour (Peeters et al. 2011). Blood was gathered in Vacutainer serum tubes (Beckton Dickinson Vacutainer Systems, United Kingdom) and centrifuged for 15 min at 1500 x g to obtain serum, which was aliquoted and immediately stored at -20 °C. Cortisol concentrations were analyzed as a single batch using a solid-phase competitive chemiluminescent immunoassay (Immulite^®^ 2000, Siemens). The serum cortisol concentration was measured using the Immulite 2000 COR kit (Siemens) following the manufacturer instructions.

### Heart rate monitoring

Interbeat interval recordings were obtained using a Polar H10 sensor system, specifically selected for its validation in equine studies (Kapteijn et al. 2022). This device allows for the automatic storage of beat-to-beat (R–R) interval recordings and associated time data within the computer for subsequent analysis. The heart rate monitor consisted of a belt designed for equine use, with electrodes positioned near the sternum. To ensure optimal conductivity, an ECG electrode transmission gel (Bleu & Marine, Bretania) was generously applied. The installation and activation of the heart rate monitor took an average of 5 min per horse. In some cases, the system started immediately. However, in other cases, it was necessary to adjust the strap multiple times and apply additional gel to receive the signal on the app. Even in the most challenging scenarios, the process never exceeded 10 min. Acclimatization to the device was unnecessary, as none of the horses’ displayed signs of discomfort or avoidant behavior during the procedure. This is likely because the horses were already highly accustomed to wearing various types of riding gear, making the strap negligible compared to the equipment they are used to. Blood sampling was performed smoothly and immediately after heart rate recording began, to check for any potential increases in heart rate. No significant heart rate changes were observed during the procedure, with variations either absent or within 2 units. Conversely, a consistent and notable increase in heart rate was observed as soon as the horses entered the experimental room.

The data collected by the Polar device was transmitted to the “HR & HRV Logger” software, allowing for real-time monitoring through a tablet running the Android operating system. Subsequently, the data was exported as a tachogram in Excel format for further analysis.

### Behavioral parameters

The ethogram utilized for analyzing horse behaviors relevant to the objectives of the current study was developed based on prior studies (Padalino et al. 2018; Schrimpf et al. 2020; Torcivia and McDonnell 2021).

In most cases, behaviors were mutually exclusive. Indeed, there were instances where behaviors could occur simultaneously, such as stress-related behaviors coinciding with other stress responses or actions directed towards strangers or the door. In such cases, both behaviors were recorded.

Given the horses’ divided attention and the large visual field (Alterisio et al. 2018), recording gaze behavior in our experimental setup presented significant challenges. Hence, we opted to record head orientation as a proxy for gazing toward a target.

Some behaviors outlined in the referenced papers were sporadically observed or noted in only a limited number of horses in our study (e.g., shaking, snuffling). Due to their limited statistical power, they were not included in our dataset and were consequently excluded from the ethogram. Similarly, behaviors not promptly associated with stress or discomfort were not considered for the study (e.g., olfactory exploration, passivity).

The resulting ethogram comprises three behavioral categories derived from specific behaviors listed in Table 1, all directly related to the objectives of the current study. Stranger-directed behavior could serve as a specific measure of the social buffer effect; door-directed behavior might indicate a desire to leave and therefore serves as an indirect measure of discomfort; and stress-related behavior indicates negative psychological responses.

**Table 1:** Ethogram employed for analyzing horse behaviors

Categories	Behaviors	Definition
Stranger-directed behaviors	Approaching the stranger	The horse goes towards the stranger from anywhere in the room. The recording of the behavior starts when the horse is focused on the stranger.
	Focused on the stranger	From a stationary position, the horse orients its head towards the stranger.
	Interaction with the stranger	The horse establishes physical contact with the stranger, e.g. rubbing, nosing, licking, biting, pushing.
Door- directed behaviors	Approaching the door	The horse goes towards the door from anywhere in the room. The recording of the behavior starts when the horse is focused on the door
	Focused on the door	From a stationary position, the horse orients its head towards the door.
	Interaction with the door	The horse establishes physical contact with the door, e.g. rubbing, nosing, licking, biting.
Stress- related behaviors	Self-grooming	Nibbling, nuzzling, or biting at a specific body area, or rubbing one part of the body against another or an object.
	Head movements	Encompassing the rotation of the head and neck along the long axis, repetitive nodding of the head and neck, flicking upward extension of the head and neck, and rhythmic side-to-side swaying of the head and neck.
	Pawing	Involves extending a forelimb forward and dragging the hoof along or above the substrate, sweeping it caudally, frequently in rhythmic series.
	Locomotion	Walking apparently without a specific target or intention.
	Snorting	Forceful expulsion of air through the nostrils preceded by a raspy inhalation sound.

To ensure the accuracy and reliability of the data, an inter-observer reliability assessment was conducted by comparing the results obtained by a second independent coder on 22% of the samples (4 videos). The agreement between coders was suitable for all variables, as indicated by Cronbach’s alpha always above 0.9.

### Statistical approach

We employed three metrics to evaluate the horses’ responses: an endocrinological metric involving cortisol levels measured in µg/dl, a physiological metric tracking heart rate inter-beat interval, and a behavioral metric focusing on the durations of horses’ responses. All behaviors indicating the same intent were summarized, and thus the behavioral categories, namely stranger-directed behaviors, door-directed behaviors and stress-related behaviors (see Table 1), served as a proxy for the statistical comparisons.

Eleven horses were included in the experiments. However, due to technical issues such as camera malfunctions and loss of signal in heart rate monitoring, data from two horses had to be excluded. Consequently, statistical analysis of heart rate monitoring and behavioral observations was conducted on 9 horses (4 females and 5 males). Given the predominantly non-normal distribution of the datasets, non-parametric approaches were adopted. The analysis of cortisol levels focused on comparing data within the horse group at the beginning (T0) and end (T1) of the test in both *isolation* (I) and *social* (S) conditions. Therefore, four variables (T0_I, T1_I, T0_S, and T1_S) were examined using the Friedman test, followed by post-hoc Wilcoxon tests for paired samples with Bonferroni correction.

While cortisol data were discrete, heart rate and behavioral data were continuously recorded, allowing observation over time. Therefore, our analysis focused on data points obtained within specific time intervals. We chose to analyze averages every 10 s, resulting in a total of 90 data points for each variable, which provides a fine detail of the temporal dynamics of these phenomena.

Data points from the isolation and social conditions were then separately analyzed using Spearman correlation, with corrections for multiple comparisons, including the timing variables. Accordingly, the variables compared in the isolation conditions were the heart rate, the stress-related behaviors, the door-directed behaviors, and the seconds. For the social condition, the stranger-directed behaviors were also included.

Finally, a generalized linear model was utilized to assess the impact of variables on heart rate. After verifying the normal distribution of residuals through visual inspection of a normal probability plot and statistical confirmation using the Shapiro-Wilk test, a linear function was selected for the target heart rate variable in both isolation and social conditions. Stress-related behaviors and door-related behaviors, along with duration in seconds, were incorporated into the isolation condition model as covariates. Additionally, stranger-directed behaviors were included as predictor variables in the social condition model. The predictor variables were tested for their main effects and their first level of interaction.

All statistical analyses were carried out using IBM SPSS statistical software version 26 (IBM Corp., Armonk, NY, USA).

## Results

### Cortisol concentration changes across isolation and social conditions

**Fig. 2:**
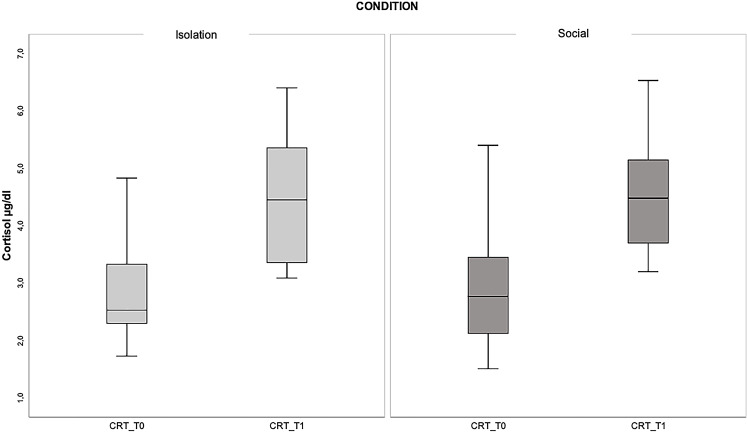
Graphics of significant differences in cortisol (CTR) levels. The box plots compare cortisol levels between T0 and T1 in both the isolation (I) and social (S) conditions. *p = < 0.05

The concentration of cortisol in the isolation (I) condition was 2.90 µg/dl ± 1.05 at T0_I and 4.40 µg/dl ± 1.16 at T1_I. In the social (S) condition it was 2.93 µg/dl ± 1.24 at T0_S and 4.50 µg/dl ± 1.03 at T1_S. The Friedman test indicated significant within-group differences (*N* = 11, χ² = 21.87; *p* < 0.001). Post hoc tests revealed no differences between the initial and final values (median T0_I 2.53 vs. median T0_S 2.77, z = 0.17, *p* = 1.00; median T1_I 4.41 vs. median T1_S 5.50, z = 0.17, *p* = 1.00). Significant differences were observed when comparing the initial and final values in both conditions. Comparison between T0 and T1 in isolation and social conditions showed identical statistical parameters (z = -3.30, *p* = 0.006). The graphical representation of cortisol analysis is reported in Fig. 2, and comprehensive statistical data are provided in Table 2.

**Table 2:** Statistical parameters for post hoc tests related to cortisol (CRT). The table displays comparisons between different samples at various time points (T0 and T1) and conditions (I = isolation; S = social), including z-scores, p-values, and corrected p-values. Significant values are highlighted in bold. The identical z-scores and p-values observed in “CRT_T0_I vs. CRT_T1_I” and “CRT_T0_S vs. CRT_T1_S” can be attributed to their shared rank distribution. Specifically, both comparisons exhibit an identical distribution of ranks, with 11 positive ranks and 0 negative ranks. This indicates that the relative ordering of observations within each pair remains consistent across both within-subject comparisons

Sample 1	Sample 2	z	*p*	*p*-corrected
CRT_T0_I	CRT_T0_S	0,165	0,869	1,000
CRT_T0_I	CRT_T1_I	-3,303	0,001	**0**,**006**
CRT_T0_I	CRT_T1_S	3,468	0,001	**0**,**003**
CRT_T0_S	CRT_T1_I	-3,138	0,002	**0**,**010**
CRT_T0_S	CRT_T1_S	-3,303	0,001	**0**,**006**
CRT_T1_I	CRT_T1_S	0,165	0,869	1,000

### Temporal correlations in heart rate and behavior during isolation and social conditions

**Fig. 3:**
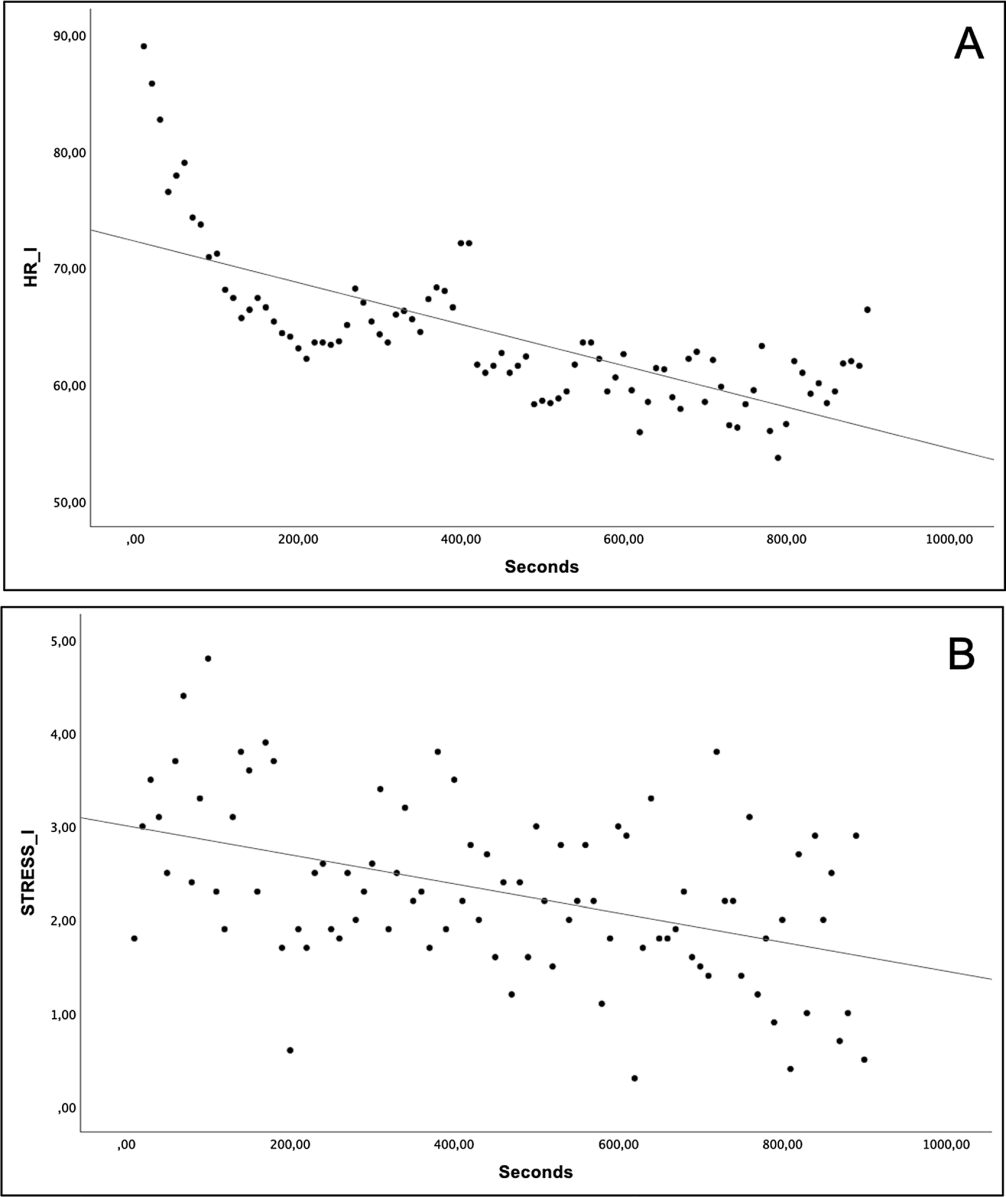
Spearman significant correlations in the isolation (I) condition. Heart rate (HR) vs. seconds (**A**); stress-related behaviors vs. seconds (**B**)

The analysis in the isolation condition revealed several correlations among the variables. Specifically, significant negative correlations were observed between heart rate (ρ = -0.775, *p* < 0.001) and stress-related behaviors (ρ = -0.420, *p* < 0.001) with seconds (Fig. 3A, B). However, no significant correlation was found between door-directed behaviors and seconds. Furthermore, heart rate demonstrated significant positive correlations with both stress-related behaviors (ρ = 0.395, *p* < 0.001) and door-directed behaviors (ρ = 0.311, *p* = 0.012).

**Fig. 4:**
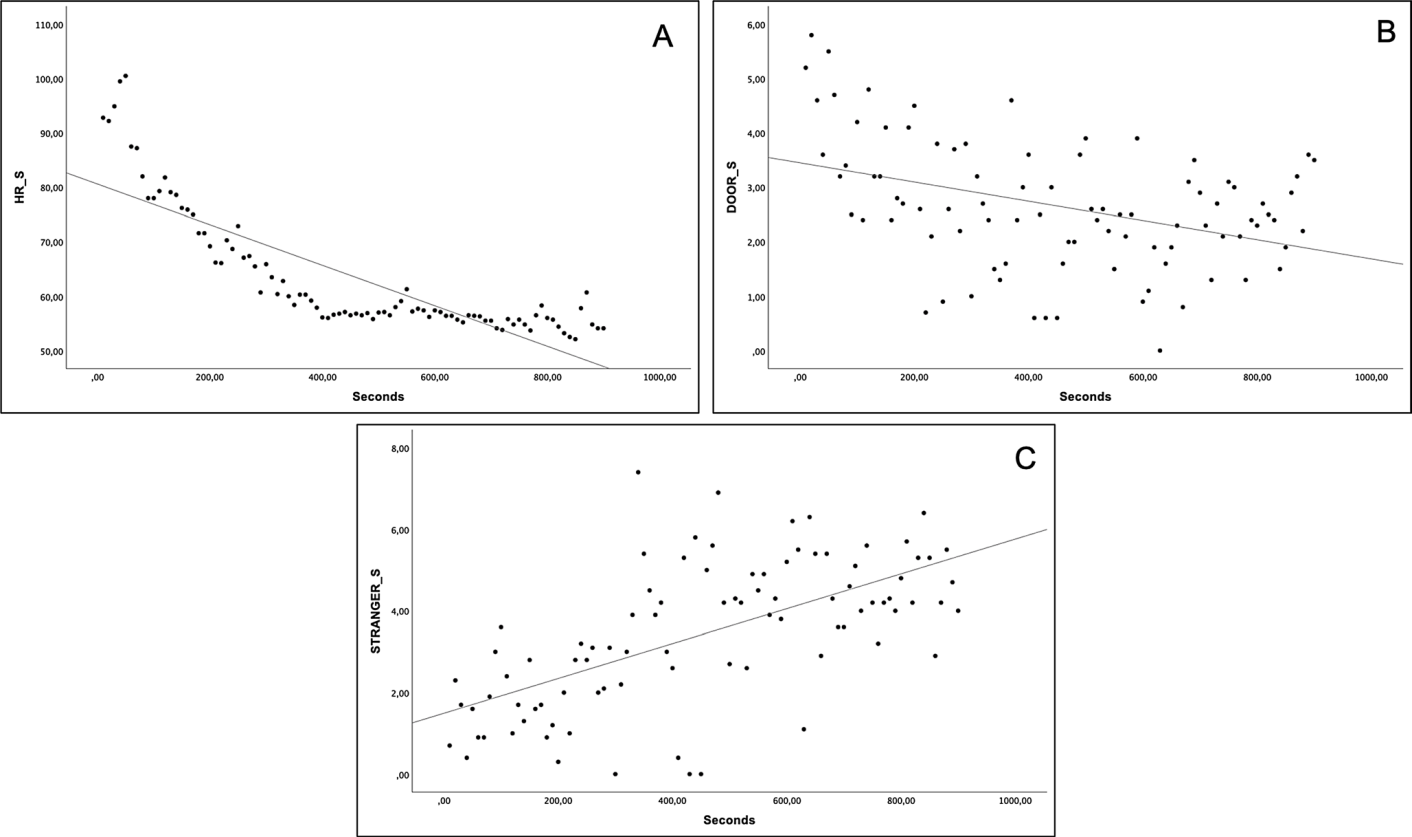
Spearman significant correlations in the social (S) condition. Heart rate (HR) vs. seconds (**A**); duration of door-directed behaviors vs. seconds (**B**); duration of stranger-directed behaviors vs. seconds (**C**)

The analysis in the social condition revealed several correlations among the variables were observed. Heart rate (ρ = -0.898, *p* < 0.001) and door-directed behaviors (ρ = -0.370, *p* = 0.002) showed negative correlations with seconds (Fig. 4A, B). Conversely, stranger-directed behaviors were positively correlated with seconds (ρ = 0.649, *p* < 0.001) (Fig. 4C). Furthermore, heart rate exhibited positive correlations with both stress-related behaviors (ρ = 0.301, *p* = 0.019) and door-directed behaviors (ρ = 0.396, *p* = 0.001), while demonstrating a significant negative correlation with stranger-directed behaviors (ρ = -0.625, *p* < 0.001). Notably, stranger-directed behaviors displayed a significant negative correlation with door-directed behaviors (ρ = -0.374, *p* = 0.001) and a trend towards a negative correlation with stress-directed behaviors (ρ = -0.263, *p* = 0.06).

### Impact of time and behavior on heart rate: generalized linear model analysis

In the isolation condition, the omnibus test conducted for the dependent variable heart rate returned a significant likelihood ratio (χ² = 79.25; *p* < 0.001). Therefore, the predictors and their interactions collectively contribute to explaining the variance in heart rate. Specifically, the analysis suggests a potential positive main effect of stress-related behaviors on heart rate (B = 3.928; *p* = 0.066), although this relationship did not reach conventional levels of statistical significance. The negative interaction between stress-related behaviors and seconds (B = -0.004; *p* = 0.032) implies that the negative effect of stress-related behaviors on heart rate diminishes over time.

The omnibus test conducted for the dependent variable heart rate in the social condition yielded significant results (χ² = 199.54; *p* < 0.001), indicating that also in this condition both predictors and their interactions collectively contribute to explaining the variance in heart rate. Stranger-directed behaviors exhibited a significant negative main effect on heart rate (B = -2.886, *p* = 0.017), indicating a decrease in heart rate with increased interaction with the stranger. Conversely, stress-related behaviors (B = 5.419, *p* = 0.010) and door-directed behaviors (B = 5.219, *p* < 0.001) demonstrated significant positive main effects on heart rate, suggesting an increase in heart rate with heightened levels of stress and discomfort. The positive interaction between seconds and stranger-directed behaviors (B = 0.005, *p* = 0.006) revealed that the effect of stranger presence on heart rate increased over time. Conversely, the negative interaction between door-directed behaviors and seconds (B = -0.009, *p* < 0.001) indicated that the effect of discomfort on heart rate decreased as time passed. Lastly, the interaction between stress-related behaviors and seconds while not reaching statistical significance (B = -0.006, *p* = 0.092), it is suggestive of a potential trend towards a reduction in the effect of stress on heart rate throughout time.

### Heart rate trends over time in isolation and social conditions

**Fig. 5:**
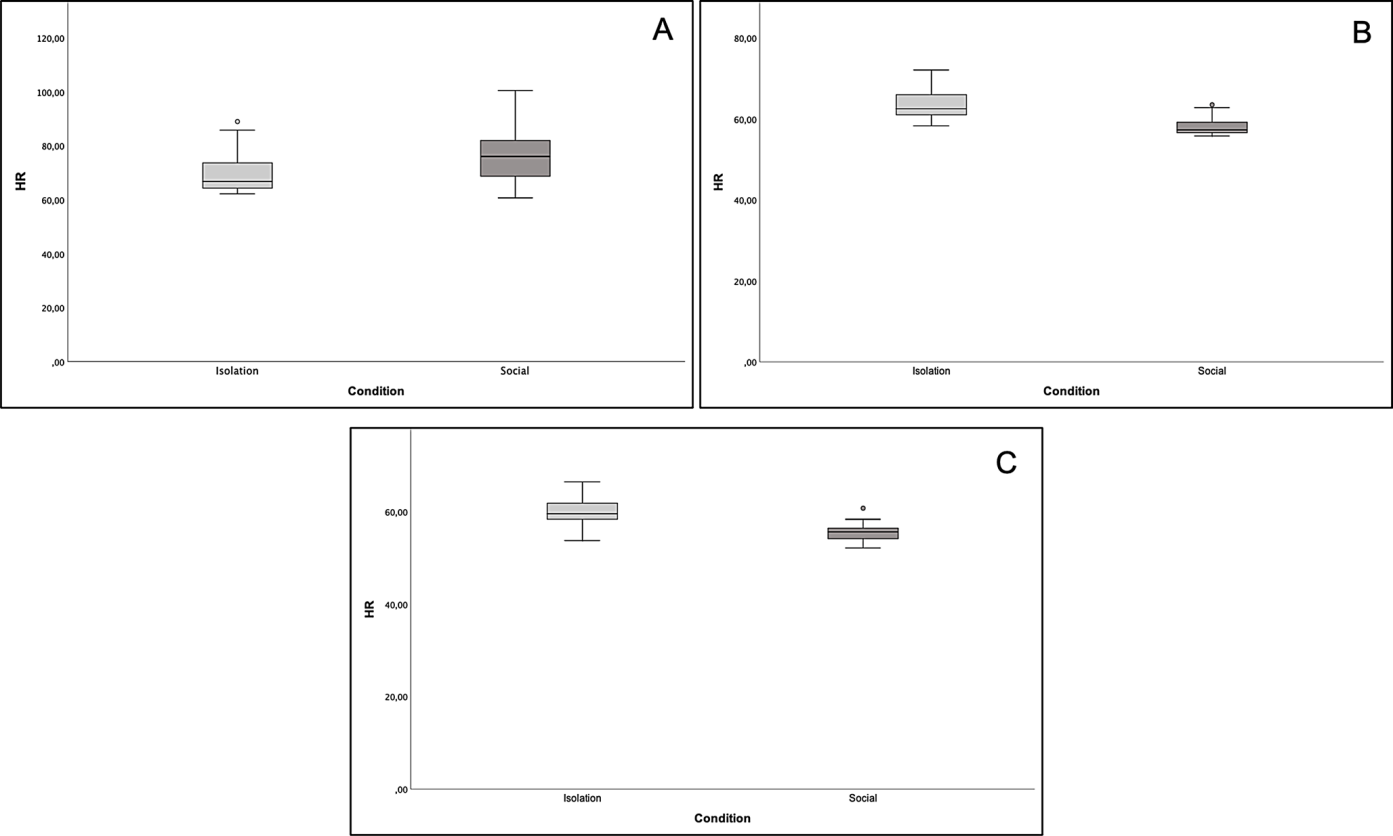
Graphical comparison of average heart rate (HR) between the isolation and social conditions across time intervals. In the first 5 min. Heart rate was significantly higher in the social condition (**A**). From minutes 6 to 10, there was a reversal trend, with heart rate significantly higher in the isolation condition (**B**). From minute 11 to 15, the pattern was the same as in B (**C**). The p-value corrected was always < 0.001

After examining the results, we noted distinct slopes in the trend lines of heart rate over time between the two conditions. Specifically, the heart rate trend line in the isolation condition had a lower slope compared to that of the social condition (compare Figs. 3A and 4A). This observation indicates that initially, the heart rate point distribution during the test was higher in the social condition than the isolation condition, while a reverted trend is present in the final part of the test. Due to this particular distribution when comparing the 90 data points between the two conditions by pairwise Wilcoxon test, no statistically significant differences are found (average heart rate: social = 63.7 ± 11.7; isolation = 64.2 ± 6.4; median heart rate: social = 57.8, isolation = 62.8; *N* = 90, z = -1.24, *p* = 0.22). Thus, although not planned, we conducted an additional analysis comparing data from the two conditions in three 5-minute intervals, which yields significant differences. Heart rate in the first 5 min was significantly higher in the social condition (average heart rate: social = 77.4 ± 10.7; isolation = 69.5 ± 7.2; median heart rate: social = 76.1, isolation = 66.8; *N* = 30; z = -4.556; *p* < 0.001; see Fig. 5A). Conversely, during the second 5 min, there was a reversal in this trend, with heart rate significantly higher in the isolation condition (average heart rate: social = 58.1 ± 2.0; isolation = 63.3 ± 3.7; median heart rate: social = 57.3, isolation = 62.5; *N* = 30; z = -4.783; *p* < 0.001; see Fig. 5B), a pattern that persisted into the last 5 min (average heart rate: social = 55.5 ± 1.8; isolation = 59.7 ± 2.7; median heart rate: social = 55.6, isolation = 59.5; *N* = 30; z = -4.433; *p* < 0.001; see Fig. 5C).

## Discussion

This study delves into the phenomenon of interspecific social buffering in horses, exploring how the presence of a human subject might impact stress responses when horses are separated from their herd and placed in an unfamiliar environment (i.e. the isolation paradigm). Three distinct metrics were employed to assess responses to a stressful event: an endocrinological metric measuring cortisol levels, a cardiac metric monitoring heart rate inter-beat interval, and a behavioral metric recording stressful and discomfort behaviors, further than the behaviors directed to the stranger in the social condition. It was anticipated that cortisol levels, heart rate, and stress-related behaviors would be lower in the presence of a stranger human subject, aligning with the social buffer hypothesis. Comparing cortisol concentrations between social and isolation conditions, we found a similar significant increase in both scenarios, with no differences observed before and after the test between conditions, suggesting that the stress response mechanism was similar regardless of the condition which contradicts our hypothesis. This finding diverges from data observed in dogs under a similar experimental paradigm. Specifically, kennel dogs with high levels of human socialization did not show increased levels of cortisol in the social condition (which involved their human caretaker), while cortisol levels increased in the isolation condition (Tuber et al. 1996). The same results were also observed in kennel dogs with low levels of human socialization when tested in social conditions with unknown people (Pinelli et al. 2023). It should be noted that in previous studies (Tuber et al. 1996; Pinelli et al. 2023), dogs experienced friendly interactions with humans, unlike the current study where the stranger was passive and provided no active emotional support to the horses. However, even with active strangers, goats, who share similar prey ecology with horses, did not benefit from human social buffering in the same way that dogs did (Scandurra et al. 2024). Thus, from a comparative perspective, it seems that while dogs may benefit from the social buffer provided by an unfamiliar human, horses may not. Considering the behavioral ecology of the two species, one might conclude that horses, as prey animals, react more fearfully in the presence of unknown humans compared to dogs, which are predators. Furthermore, in studies on dogs, humans interacted with the animals, unlike in our study, where the human avoided visual contact with the horses. This lack of interaction may have served as an additional stressor for the horses alongside social isolation.

Indeed, when we analyzed data over time, a different perspective emerged. The results from the Generalized Linear Model highlight that, while stress and discomfort generally decrease in both conditions, the stranger’s presence played a pivotal role. Specifically, there was a significant decrease in heart rate with increased interaction with the stranger, and the positive interaction between time and stranger-directed behaviors indicated that the stranger’s influence on heart rate strengthened over time. In the initial 5-minute period, heart rate during the test was significantly higher in the social condition compared to isolation. However, this pattern reversed in the subsequent two 5-minute intervals. This intriguing finding suggests that horses initially experience heightened stress upon encountering a stranger, potentially compounding the stress related to isolation. The initial approach with the stranger may have caused the horse to feel anxious due to its prey nature, which makes the species more cautious. However, as time progressed and the horse realized that the human posed no threat, it began to effectively benefit from the stranger’s presence. Thus, our findings support the hypothesis that a passive stranger can provide a social buffer for horses during stressful conditions, with potentially increasing benefits. This interaction facilitated faster heart rate recovery, as indicated by the steeper slope of the regression line in the social condition. The latter result aligns with previous studies showing that horses experience faster heart rate recovery when they have intraspecific social companions during stress tests (Ricci-Bonot et al. 2021), suggesting that social buffering plays a significant role in horses’ physiological recovery after unexpected events in both intraspecific and interspecific interactions.

The observed trend can also explain the discrepancy with cortisol data. The cortisol concentration in horses rises rapidly in circulating blood in response to stressors (Irvine and Alexander 1994; Kang and Lee 2016). In humans, studies highlight that the recovery of cortisol levels to baseline following a stressful event depends on variables such as the stressor’s nature, individual differences, and overall health, and most importantly, can potentially span hours (Dimitrov et al. 2018). In the context of our testing sessions lasting 15 min, the cortisol increase due to initially perceived stress in horses may still be detectable at the session’s conclusion, reflecting stress responses from several minutes earlier. This temporal delay suggests that cortisol may not effectively measure a social buffering effect immediately after brief stressful events. Nevertheless, cortisol remains a valuable tool for assessing social buffering effects when social interactions impede cortisol elevation, as observed in dogs (Tuber et al. 1996; Pinelli et al. 2023).

Overall, this study highlights the complex and multifaceted nature of stress responses in horses, emphasizing the need for comprehensive approaches that integrate endocrinological, physiological, and behavioral metrics.

The current study focused on horses that are well-socialized with unfamiliar people, so it remains uncertain whether horses can experience a social buffering effect from humans if they were to be mistreated. Furthermore, it is important to assess the impact of the stressor itself. Ricci-Bonot et al. (2021) showed that while a companion reduced behavioral responses in the presence of a novel object, it did not have the same effect during an umbrella opening test, which horses perceive as more threatening. This indicates that the effectiveness of the social buffering effect may vary depending on the nature of the stressful event. Further research with diverse stressors and contexts is needed to better understand the role of humans as social buffers for horses. In particular, it will be crucial to investigate different stress-inducing scenarios with and without human presence to comprehensively explore how human interaction can alleviate stress in horses.

## Data Availability

The datasets generated and analyzed during the current study are available from the corresponding author on reasonable request.
